# Gain-Framed Messaging for Promoting Adult Sport: Examining the Effects of Efficacy-Enhancing Information

**DOI:** 10.3389/fpsyg.2019.00431

**Published:** 2019-03-08

**Authors:** Meagan Littlejohn, Bradley William Young

**Affiliations:** Faculty of Health Sciences, University of Ottawa, Ottawa, ON, Canada

**Keywords:** gain-framed messaging, self-efficacy, adult sport promotion, outcome expectancies, sport behavior, masters sport promotion

## Abstract

Sport is a potential venue for more middle-aged adults to engage in sufficient physical activity for health benefits. Little is known about whether messaging interventions can motivate sport activity. This experiment tested the impact of gain-framed messaging (i.e., information about the benefits of doing adult sport) based on the inclusion (or lack thereof) of efficacy-enhancing information. Adults (30–69 years-old) were randomly assigned to experimental (a 4-min online video of “Gain-framed messages alone,” or “Gain-framed plus efficacy-enhancing messages”) or control conditions. Participants (*N* = 232; 62.5% female) completed baseline measures for intentions, barrier and scheduling self-efficacy, outcomes expectancies (OEs), sport behavior and moderate-to-vigorous physical activity, received their condition 1-week later, reported measures immediately after, and 1-month later. Results showed no differences between the experimental conditions, indicating there was no advantage of supplemental efficacy-enhancing information compared to gain-framed messages alone. When the two messaging groups were collapsed, they showed significant increases for OEs related to travel, social affiliation, and stress relief immediately following experimental exposure, compared to the control group. Overall, there were few benefits attributed to messaging and no effects on self-reported sport registration or sport behavior. Discussion focuses on future messaging considerations that may more effectively motivate adult sport participation.

## Introduction

Understanding how to best communicate information about the benefits of regular physical activity (PA) and the conditions under which such information persuades people to become more active is important for interventions in health. The communication of gainful messages and health outcomes relating to adult exercise, fitness and PA has been a popular area of study (Brawley and Latimer, 2007); however, strategies for specifically communicating gainful attributes of adult sport have received almost no attention. Considering that sport may be a viable conduit for community health for an ever-increasing segment of our population (Khan et al., 2012), the current experiment investigated gain-frame *messaging* applied to adult sport.

Messaging is a technique for framing the presentation of persuasive/motivating messages to stimulate engagement in desirable behaviors (Rothman and Updegraff, 2010). Unlike loss-framed messages (LFM) that highlight costs of not performing a target activity, gain-framed messages (GFM) emphasize anticipated favorable circumstances associated with performing it. GFM are generally more effective when promoting PA (O'Keefe and Jensen, 2007; Gallagher and Updegraff, 2012), with evidence showing their effectiveness for changing psychological and behavioral outcomes (e.g., Brawley and Latimer, 2007; Berenbaum and Latimer-Cheung, 2014). In light of the fact that almost all GFM research in PA has been focused on exercise, fitness, and rehabilitative domains, this investigation sought to examine various messaging formats for promoting adult sport to middle-aged adults.

Researchers have stressed the importance of understanding various conditions that enhance messaging effectiveness for PA (Latimer et al., 2010; Hatchell et al., 2013). GFM have been shown to effectively communicate information to increase awareness/knowledge of anticipated benefits of PA, though this has not always been enough to stimulate actual behavior change in recipients. For instance, Cavill and Bauman (2004) discussed the importance of increasing knowledge and awareness for eventual behavior change, yet noted that self-efficacy needed to be additionally targeted to motivate behavior change. Furthermore, paired messaging interventions, wherein GFM were coupled with approaches that targeted additional cognitive correlates of behavioral change, appeared to have greater capability in eliciting behavioral outcomes (Latimer et al., 2008; Sweet et al., 2014). In particular, Latimer et al. (2010) suggested greater promise for interventions that pair content targeting self-efficacy (SE) beliefs with GFM. The current study investigated the pairing of GFM and SE conditions for the promotion of adult sport.

Few randomized-controlled experiments have systematically analyzed effects of supplementing GFM with an efficacy enhancement condition. Graham et al. (2006) examined the effects of pairing SE-enhancing information with GFM about the ability of exercise to decrease the risk of colon cancer in a sample of low-active adults (*M age* = 43). Compared to a control group (that received information about diet and cancer), experimental participants reported higher response efficacy and intentions to exercise. Latimer et al. (2008) investigated the relative effectiveness of GFM and LFM in adults (19–69 years old). Participants in both conditions also received message content designed to increase SE for overcoming barriers to PA. Results showed an advantage for the GFM condition in increasing intentions to be active, SE, and PA compared to LFM. Sweet et al. (2014) examined GFM paired with efficacy-enhancing messages about action planning for PA among inactive adults (*M age* = 41). This paired messaging group was equally likely as a GFM-alone group to create action plans, but their plans were significantly more detailed. These studies suggested that paired messaging conditions enhancing SE could possibly facilitate positive change on key outcomes. However, none of these studies systematically contrasted the effects of a paired (GFG+SE) condition to each of a GFM-alone and a control condition, to more systematically tease out contributions.

Only one study has used a messaging framework to promote sport participation (Lithopoulos et al., 2015). Researchers assessed the effects of a GFM intervention highlighting benefits of adult sport to 40–59 year-olds who were not regularly doing sport. Participants who viewed a GFM video, as opposed to a comparison group (quiz about sport and PA), elaborated significantly more about a hoped-for sport self on themes that were consistent with the GFM video. GFM recipients also requested significantly more sport-related newsletters, and were more likely to have registered for a sport program 1 month later. In a posteriori analyses, Lithopoulos and Young (2016) discussed how their protocol inadvertently created “paired” and “unpaired” conditions with the addition of a possible selves protocol (PSP), and how there were enhanced benefits for GFM+PSP recipients compared to those who received GFM alone, whereby they requested more newsletters and reported higher registration rates. This study showed some promising results in sport, and trends indicating an advantage for GFM in a paired condition. There remains a need to examine whether a GFM and SE-enhancing pairing might promote different outcomes for attracting middle-aged persons to sport.

## Objectives and Hypotheses

The current experiment aimed to answer three questions: (1) What are the psychological effects (on outcome expectancies, intentions, SE) of a GFM-alone condition compared to a paired (GFM+SE-enhancing information) condition?; (2) How are sport behavior and moderate-to-vigorous physical activity (MVPA) impacted after exposure to GFM-alone compared to GFM+SE?; (3) What are the psychological and behavioral effects of receiving *either* messaging condition (GFM-alone or GFM+SE) compared to a control condition? These questions were examined immediately after exposure and at 1-month post intervention. The hypotheses were: the GFM+SE condition would result in higher SE beliefs than GFM-alone; the GFM+SE condition would report higher intentions, rates of sport registration, sport activity and MVPA, and be more likely to request a sport-related newsletter than GFM-alone; GFM+SE and GFM-alone would report increases in outcome expectancies (OEs); GFM+SE and GFM-alone would show greater increases in psychological and behavioral outcomes than Control.

## Method

### Participants

Participants were recruited from various sources in Canada, including at community centers and at youth sport events, and via social media platforms (e.g., Facebook) and online boards (e.g., Kijiji). All participants provided informed consent and partook voluntarily. A total of 603 participants initially completed the first of three surveys. Of these, 475 (61.9% female; *M age* = 45.93, *SD* = 7.92) met inclusion criteria—they were 30–69 years-old and did not perceive adult sport to be risky (≤5 on a scale from 1— “not at all risky” to 7 — “extremely risky”). Pragmatically, it does not make sense to test gain-frame messaging (in essence, a persuasive advertisement) for a target audience that reports that prospective sport activity is inherently personally risky. Thus, prior to randomized assignment, the decision was made to not include participants who reported 6 or 7 on the perceived risk scale^1^.

### Procedure and Data Collection

All protocol were approved by the research ethics board at the host institution. All survey items, as well as the messaging interventions, were implemented using FluidSurveys.com.

#### Time 1

Recruited participants were emailed the link for the first online survey. They completed demographic and inclusion criteria measures, questions assessing stage of change (Prochaska et al., 1992) with respect to sport, health status (“Do you currently consider yourself healthy enough to regularly participate in sport?”; yes/no), and sport engagement during youth (yes/somewhat/no). They then completed dependent measures for weekly sport behavior, MVPA, intentions, barrier SE, scheduling SE, and OEs.

*Typical weekly sport behavior* in the past month was assessed using a modified version of the Short Questionnaire to Assess Health Enhancing Physical Activity (SQUASH; Wendel-Vos et al., 2003). Participants listed each of the sports they engaged in during an average week, then reported how many times weekly and how much time (in minutes) they attributed to each, as well as whether engagement was of light, moderate or intense effort^2^. These scores were then used to dichotomously code participants as either “yes” (participating in sport) or “no.” *MVPA* was assessed using the Godin Leisure Time Exercise Questionnaire (GLTEQ; Godin and Shephard, 1985), which measured engagement in a typical 7-day period without distinguishing sport from exercise/fitness activities.

*Intentions* were measured using five items on a 7-point Likert scale previously used to assess messaging outcomes (Lithopoulos et al., 2015). For example, items asked “How likely is it that you will participate in sport activity sometime soon?” and “If faced with the decision to begin regular participation in sport today, how likely is it that you would do so?” This scale had high internal consistency in our sample (α = 0.92).

*Barrier SE* was assessed using five items, each asking about a barrier to sport participation (i.e., lack of time, lack of motivation, negative attitude, lack of sport facilities/opportunities, lack of encouragement from others). As per Bandura (2006) guidelines, participants indicated from 0 to 100 (in increments of 10) their confidence in their ability to overcome each barrier. For example, they judged their confidence to regularly participate in sport (defined as three of more times per week for at least 150 min) “When I have other time commitments.” The 5-item scale showed acceptable reliability (α = 0.77). *Scheduling SE* was measured to derive an index of confidence in one's ability to schedule increasing amounts of sport activity. After the prompt, “Assuming you are motivated, in the next month, how confident are you that you can fit at least 30 min of moderate-to-heavy intensity sport participation into your weekly schedule,” participants responded on a Likert scale (1 = “not at all confident” to 7 = “completely confident”) for different options (once; twice; three; four; five, or more times per week; see Arbour-Nicitopoulos et al., 2009, for validity of this protocol with adults). The scale had strong reliability in our sample (α = 0.93).

*OEs* were assessed to derive an index of beliefs about the likelihood that regular sport participation would result in favorable outcomes (Gellert et al., 2012). Participants judged items for nine gainful outcomes (each one matched a message in our GFM): *optimal health (OE-Health)*; *delayed effects of aging (OE-Aging)*; *social affiliation (OE-Friends)*; *fun/enjoyment (OE-Fun)*; *stress relief (OE-Stress relief)*; *improvement of physical capabilities (OE-Physical capabilities)*; *thrills/excitement (OE-Thrills)*; *achievement of competitive goals (OE-Goals)*; *travel opportunities (OE-Travel)*. For example, in response to “Regularly participating in sport can give me the opportunity to make new friends,” participants made judgments on a Likert scale (1 = “strongly disagree” to 5 = “strongly agree”). Pearson correlations between the nine OE items at T1 (all <0.70) revealed that multicollinearity was not a concern; thus, each OE was treated as a separate dependent variable.

A possible covariate, *attitudes toward adult sport*, was assessed with a semantic differential scale (Berenbaum and Latimer-Cheung, 2014) modified for sport. Participants made Likert scale responses to the prompt, “For me, regularly participating in sport as an adult would be” according to seven anchors: good—bad; beneficial—harmful; valuable—worthless; enjoyable—unenjoyable; pleasant—unpleasant; interesting—boring; relaxing—stressful. The scale had strong reliability (α = 0.90).

#### Time 2

After completion of the T1 survey, each consecutive suite of five responders that met inclusion criteria for age and perceived risk was assigned randomly to groups in a 2-2-1 fashion (i.e., 2 respondents to GFM-alone, 2 to GFM+SE, 1 to the control). Participants were sent an email inviting them to open a web-link to view their intervention (GFM-alone or GFM+SE). Control participants did not receive any messaging; they opened their web-link and completed the survey measures.

#### GFM-Alone

Participants received a 4-min narrated PowerPoint Presentation (PPT) video with nine consecutive GFM, each highlighting a benefit of being involved in adult sport, followed by five neutral slides. Messages were tailored to each person's sex by assigning a female/male narrated voice. The GFM replicated the promoted benefits from Lithopoulos et al. (2015), taken from research on involvement opportunities gained through adult sport participation (Young et al., 2015; see Appendix A in Supplementary Material for messages). There were neutral slides that were added containing factual/historical information about adult sport to ensure both experimental conditions received the same number of messages in a video of the same length. Message order was randomized, and participants were not able to pause the video such that messages were not repeated, with equal time per message.

#### GFM+SE

Participants received a 4-min narrated PPT video. The first nine messages were the GFM in a randomized order, followed by five randomized SE messages, each designed to increase SE to regularly participate in sport when faced with a common barrier, by suggesting a technique to overcome each barrier. After extensively reviewing the most common barriers to adult sport (e.g., Cardenas et al., 2009), five SE-enhancing messages for navigating barriers were created, specifically pertaining to: lack of time; lack of motivation; negative attitudes; lack of sport facilities/opportunities nearby; lack of encouragement from others. Each message comprised phrasing reflecting vicarious (e.g., “other adults just like you have done it”) and verbal persuasive (“you too can do it”) efficacy enhancement sources (Bandura, 2001; see Appendix B in Supplementary Material).

#### Immediately Following Video Exposure

Experimental participants were asked to type out two main themes they recalled being presented in the messages. The primary investigator reviewed the responses from each participant (i.e., what they had typed in an open-ended box) and coded distinct themes, enumerated them (0, 1, or 2 or more themes being recalled). This served as a manipulation check; 12 participants (*GFM-alone* = 7; *GFM*+*SE* = 5) could not recall two themes and were excluded from the analyses.

#### Time 2 Survey Measures

All participants reported intentions, barrier SE, scheduling SE, and OEs. They were also asked whether they would like to request *a newsletter relating to adult sport opportunities* in their community (yes/no), which is a proxy measure for interest and information-seeking (Lithopoulos et al., 2015).

#### Time 3

Four weeks later, participants reported intentions, barrier SE, and scheduling SE, as well as weekly sport behavior, weekly MVPA, whether they had *registered for a sport program* (e.g., an organization, team, club; yes/no), and whether they had *registered for a sport event* (e.g., a local 10 km race; yes/no) within the past month.

## Analyses and Results

### Preliminary Analyses

Each dependent variable at baseline was inspected for normality. *OE-Health* showed evidence of skewness (−2.27) and kurtosis (4.90), and the *OE-Aging* had some kurtosis (2.20), but all other variables showed skewness and kurtosis values < ±2 (Field, 2005). Analyses were performed to inspect outliers all on dependent variables at T1. The MVPA scores of three participants revealed them as extreme outliers (>3.29 *SD*); they were excluded from further analyses. Baseline equivalency tests were performed to inspect group differences at T1 for participants who completed the entire study. The groups were not significantly different (see Table 1), except more control participants perceived themselves as *not* healthy enough to regularly participate in sport (*p* < 0.001). Although some participants judged themselves not healthy enough for sport, their GLTEQ data showed high activity levels in other forms of PA. As such, there was little concern about risk, so the decision was made to conduct and report all main analyses with the total sample, irrespective of their health status judgments To confirm our results were replicated with the smaller cohorts that reported being “healthy enough” to regularly participate in sport, all analyses were re-run with only those participants in each group. Results were the same for the healthy-enough sample, as were effect sizes, with two exceptions that are identified in subsequent endnotes^3^. With respect to the dependent variables (see Tables 2, 3 for descriptives), there were only two between-group differences at T1: *OE-Fun* (*p* = *0.0*2) and *OE-Physical capabilities* (*p* = *0.0*2).

**Table 1:** Descriptive statistics for demographic and screening variables at baseline.

	**GFM-alone**	**GFM+SE**	**Control**
N	79	84	69
Mean age (*SD*)	47.3 (8.13)	47.9 (7.32)	44.7 (10.3)
Gender (male, female, other) Mean risk perception (*SD*)	30, 48, 1 2.62 (1.43)	32, 50 (2 missing) 2.55 (1.43)	47, 21 2.93 (1.43)
**PERCEIVED HEALTH STATUS**			
Healthy (%)	87.3	85.5	63.8
Not healthy (%)	12.7	14.5	36.2
**WEEKLY SQUASH SPORT STATUS**			
Active in sport (%)	32.9	35.7	24.6
Not active in sport (%)	67.1	64.3	75.4
**YOUTH SPORT PARTICIPATION**			
Yes (%)	58.2	57.1	58.0
Somewhat (%)	15.2	11.9	8.7
No (%)	25.3	25.0	31.9
Missing data (%)	1.3	6.0	1.4
**STAGE OF CHANGE**			
1–Pre-contemplation (%)	43.1	36.9	53.6
2–Contemplation (%)	13.9	14.3	13.2
3–Preparation (%)	6.3	9.5	1.4
4–Action (%)	5.1	3.6	2.9
5–Maintenance (%)	29.1	35.7	27.5
Missing data (%)	2.5	0.0	1.4

**Table 2:** Descriptive statistics for outcome expectancy variables at multiple time points.

		**Time 1**		**Time 2**	
**Group**		**Mean**	***SD***	**Mean**	***SD***
GFM-alone	OE-Health	4.46	1.04	4.51	0.87
	OE-Aging	4.19	1.02	4.35	0.77
	OE-Friends	3.95	1.04	4.09	0.87
	OE-Fun	4.01	1.17	3.97	1.00
	OE-Stress relief	3.90	1.13	3.82	1.13
	OE-Physical capabilities	3.95	1.10	4.15	0.92
	OE-Thrills	3.53	1.06	3.71	1.09
	OE-Goals	3.24	1.17	3.58	1.12
	OE-Travel	2.63	1.35	3.04	1.16
GFM+SE	OE-Health	4.59	0.84	4.57	0.91
	OE-Aging	4.41	0.93	4.30	0.96
	OE-Friends	3.94	1.05	4.17	0.82
	OE-Fun	4.34	0.98	4.14	0.96
	OE-Stress relief	4.29	0.96	4.19	0.94
	OE-Physical capabilities	4.34	0.83	4.29	0.80
	OE-Thrills	3.76	1.15	3.76	0.94
	OE-Goals	3.52	1.11	3.57	1.15
	OE-Travel	2.77	1.20	3.10	1.17
Control	OE-Health	4.51	0.87	4.45	0.82
	OE-Aging	4.25	1.01	4.24	0.93
	OE-Friends	4.09	0.90	3.91	0.94
	OE-Fun	3.88	1.11	3.75	1.18
	OE-Stress relief	4.07	1.09	3.72	1.18
	OE-Physical capabilities	3.96	1.06	4.03	0.97
	OE-Thrills	3.45	1.19	3.32	1.11
	OE-Goals	3.31	1.22	3.34	1.22
	OE-Travel	2.91	1.15	2.69	1.16

**Table 3:** Descriptive statistics for self-efficacy, intentions and MVPA variables at multiple time points.

		**Time 1**		**Time 2**		**Time 3**	
**Group**	**Variable**	**Mean**	***SD***	**Mean**	***SD***	**Mean**	***SD***
GFM-alone	Barrier SE	50.4	23.1	49.5	20.6	52.9	19.2
	Scheduling SE	3.63	1.86	3.35	1.63	3.42	1.73
	Intentions	4.01	2.01	3.88	1.80	3.83	1.83
	MVPA (GLTEQ)	23.6	18.3	–	–	23.6	17.6
GFM+SE	Barrier SE	52.2	23.0	51.8	25.5	52.5	23.0
	Intentions	3.82	1.73	3.82	1.73	3.99	1.85
	Scheduling SE	3.76	1.85	3.68	1.65	3.66	1.70
	MVPA (GLTEQ)	27.9	19.2	–	–	29.4	20.6
Control	Barrier SE	44.6	25.9	46.8	22.7	44.1	22.6
	Scheduling SE	3.34	1.98	3.44	1.71	3.43	1.88
	Intentions	3.93	2.06	3.70	1.93	3.94	1.99
	MVPA (GLTEQ)	26.8	22.6	–	–	24.7	22.5

*Mean barrier SE scores were 0–100, intentions and scheduling SE Likert-scale scores ranged from 1 to 7, and MVPA scores were composite scores from the GLTEQ, with higher values representing greater activity*.

Bivariate correlations were conducted to determine potential covariates (Tabachnick and Fidell, 2012). T1 *attitudes toward sport* correlated strongly with *intentions* (*r* = 0.64) and moderately with the *OEs* (ranged from 0.15 to 0.54); considering theoretical rationale that attitudes toward an activity affect intention (Azjen, 1991), *attitudes* became a covariate for all analyses pertaining to intentions and OEs. Due to moderate-to-strong correlations, T1 *intentions* were identified as a covariate for analyses pertaining to *barrier SE* (*r* = 0.33), *MVPA* (*r* = 0.53), and *scheduling SE* (*r* = 0.59).

#### Attrition Analyses

A total of 475 participants completed the T1 survey and were eligible for the study, 244 completed the T2 survey, and 232 completed T3. Tests were performed, for the entire sample and on a within-group basis, to compare T1 characteristics between participants that completed the entire study and those that dropped out at some point. Analyses for the entire sample showed differences between “completers” and “drop-outs” for *group assignment, age, weekly sport behavior*, and six *OEs* (all *p*s < 0.03). GFM-alone (drop-outs = 41.2%) and GFM+SE (39.5%) participants were more likely than control participants (19.3 %) to drop-out. Completers were older than drop-outs within the GFM+SE group (completers: *M* = 47.9, drop-outs: *M* = 44.8) and within the GFM-alone group (completers: *M* = 47.3, drop-outs: *M* = 44.8). Drop-outs were likelier to report some current sport-related participation (74.8%) than completers (31.5%), which also held true in each group. Control group completers were likelier to have not participated in youth sport (no = 32.5%*;* somewhat participated = 8.8%*;* participated in youth sport = 58.5%) than drop-outs (no = 4.5%*;* somewhat = 27.3%*;* participated in youth sport = 4.5%). For the entire sample, and within the Control group, drop-outs reported higher OEs for Fun, Stress relief, Physical capabilities, Thrills, Goals, and Travel than completers at T1.

### Main Analyses

#### Did the GFM-Alone and the GF+SE Conditions Elicit Different Effects?

##### Immediate effects

A series of Group × Time (T1, T2) repeated-measures analysis of covariance (RM-ANCOVAs) compared trends between the experimental groups in *OE* responses. No significant interaction effects were found for any of the separate analyses for the nine *OE* variables (*Health, p* = 0.75; *Aging, p* = 0.10; *Friends, p* = 0.97; *Fun, p* = 0.23; *Stress relief, p* = 0.51; *Physical capabilities, p* = 0.14; *Thrills, p* = 0.28; *Goals, p* = 0.11; *Travel, p* = 0.70). A binary logistic regression tested if assignment to one of the groups better predicted *requests for a sport-related newsletter* (controlling for T1 intentions). The model was not significant, $X_{\left(2\right)}^{2}$ = 1.91, *p* = 0.38, Nagelkerke *R*^2^ = 0.01.

##### Outcomes across three time points

Separate Group × Time (T1, T2, T3) RM-ANCOVAs were performed for *intentions, barrier SE* and *scheduling SE*. No significant interactions resulted: *intentions* [*p* = 0.65, $\eta_{\text{p}}^{2}$ = 0.00], *barrier SE* (*p* = 0.36, $\eta_{\text{p}}^{2}$ = 0.01), *scheduling SE* (*p* = 0.54, η^2^_*p*_ = 0.01). A Group x Time (T1, T3) RM-ANCOVA was conducted for *MVPA* scores, resulting in a non-significant interaction (*p* = 0.88, $\eta_{\text{p}}^{2}$ = 0.00). Finally, a chi-square tested group differences in the frequency of *weekly sport activity* designations (yes/no) at T3, using T1 sport activity categorization (yes/no) as a control variable. There was no group difference, [$X_{\left(1\right)}^{2}$ = 2.0, *p* = 0.15, Cramer's V = 0.12; % active in sport: GFM+SE = 29.9%; GFM-alone = 41.9%. Results showed that those in both experimental groups that were not active in sport at T1 were equally as likely to report sport activity at T3, [$X_{\left(1\right)}^{2}$ = 0.94, *p* = 0.33, Cramer's V = 0.10; % active in sport: GFM+SE = 9.5%; GFM-alone = 16.7%.

##### Outcomes measured only at 1-month follow-up

For participants not active in sport at T1, separate binary logistic regressions examined if assignment to one group better predicted *registration in a sport program* and *registration in a sport event*, while controlling for T1 intentions. Although the model for program registration was significant, $X_{\left(2\right)}^{2}$ = 11.29, *p* < 0.001, Nagelkerke *R*^2^ = 0.15, *post-hoc* examination indicated “Group” did not contribute to variance in registration frequency (*p* = 0.64, *B* = 1.2). The model for event registration was non-significant, $X_{\left(2\right)}^{2}$ = 1.87, *p* = 0.39, Nagelkerke *R*^2^ = 0.03.

#### Comparing a Collapsed Intervention Group to the Control Group

Since the experimental groups acted similarly, the groups were collapsed (called the “Intervention” group) and submitted to the same aforementioned series of analyses contrasting the Intervention to the Control group.

##### Immediate effects

**Figure 1:**
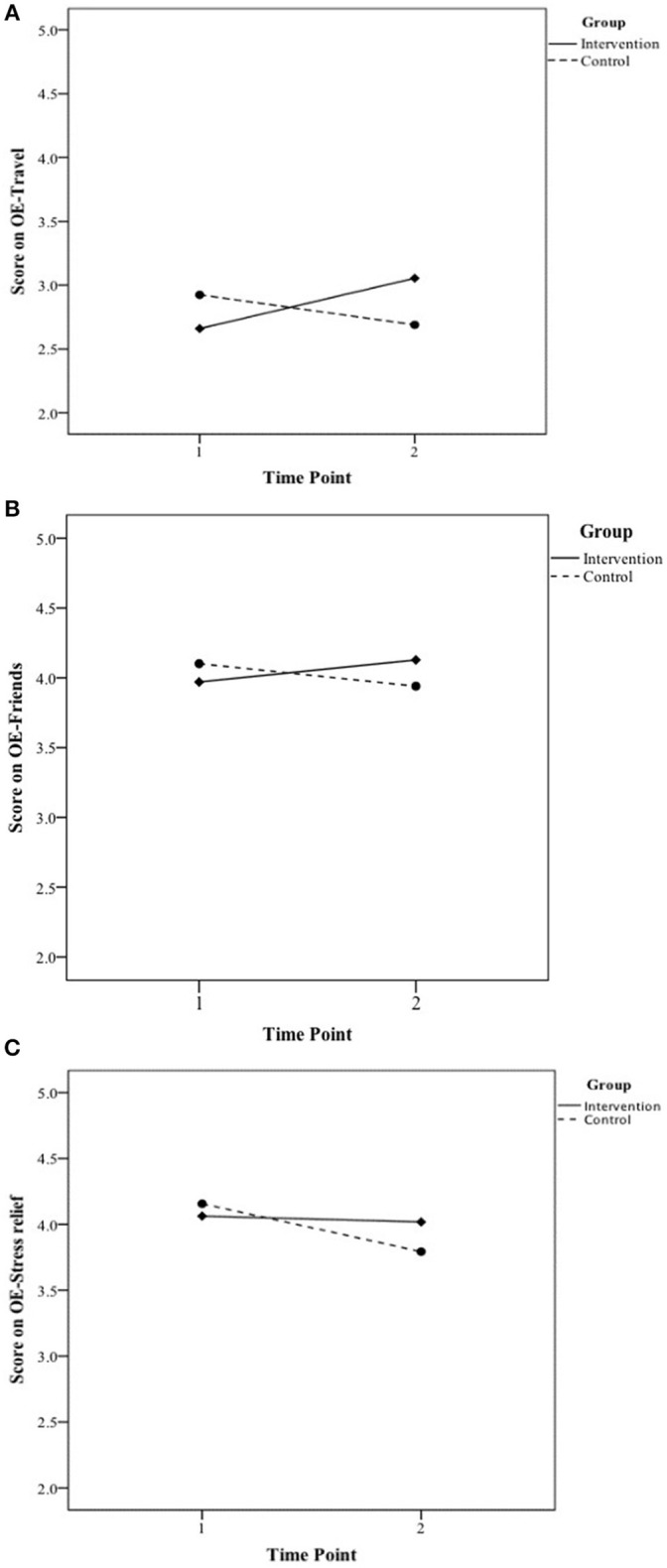
Results for significant group by time interactions found when comparing a collapsed Intervention group (comprising GFM-alone and GFM+SE) to the Control group. **(A)** Represents expectancies to experience opportunities to travel, **(B)** Represents expectancies to have opportunities to be with friends, and **(C)** Represents expectancies to relieve stress via regular adult sport participation.

Results revealed interaction effects for *OE-Travel* [*F*_(1, 219)_ = 12.3, *p* < 0.001, η^2^_*p*_ = 0.05], *OE-Friends* [*F*_(1, 217)_ = 5.30, *p* = 0.02, $\eta_{\text{p}}^{2}$ = 0.02], and *OE-Stress relief* [*F*_(1, 217)_ = 3.78, *p* = 0.05, $\eta_{\text{p}}^{2}$ = 0.01] (see Figures 1A–C). *Post-hoc* pairwise comparisons revealed the Intervention group reported increased expectations of sport to provide travel opportunities at T2 compared to T1 (*p* < 0.001, η^2^_*p*_ = 0.07), and they had higher OE-Travel than the Control at T2 (*p* = 0.03, η^2^_*p*_ = 0.02). *Post-hoc* comparisons for *OE-Friends* showed the Intervention group increased their expectations about sport providing opportunities to make new friends at T2 compared to T1 (*p* = 0.03, η^2^_*p*_ = 0.02). The Control group did not change over time (*p* = 0.17), and the Intervention and Control groups were not different at T2 (*p* = 0.14). Pairwise comparisons for *OE-Stress relief* revealed the Control group decreased their expectations from T1 to T2 (*p* < 0.001, $\eta_{P}^{2}$ = 0.03), whereas the Intervention group did not decrease over time (*p* = 0.61); the groups were not different at T2 (*p* = 0.12). There were no significant interactions for the other OEs^4^. Generally, the Intervention group consistently trended positively in OEs over time, while Control group values remained constant or slightly decreased (Table 2).

With respect to *requests for a sport-related newsletter*, the model was significant, $X_{\left(2\right)}^{2}$ = 10.94, *p* < 0.001, Nagelkerke *R*^2^ = 0.06; however, “Group” did not contribute to variance in frequency of requests (*p* = 0.15, *B* = 0.65).

##### Outcomes across three time points

Results showed non-significant interactions for *intentions, barrier SE* and *scheduling SE* (all *ps* > 0.45). There was a main effect for time for *scheduling SE* [*F*_(2, 316)_ = 2.94, *p* = 0.05, $\eta_{\text{p}}^{2}$ = 0.01], whereby levels decreased significantly at successive time points for all participants. Results for *MVPA* showed an interaction approaching significance [*F*_(1, 180)_ = 3.13, *p* = 0.07, η^2^_*p*_ = 0.01]^5^. Although none of the pairwise *post-hoc* tests reached significance (all *p*s > 0.12), the Control group showed decreased trends for MVPA from T1 to T3, while the Intervention group increased slightly. There was no group difference for frequency of *weekly sport behavior* at T3, $X_{\left(1\right)}^{2}$ = 0.85, *p* = 0.35, Cramer's V = 0.06; % active in sport: Intervention = 35.7%; Control = 28.8%. Among participants who were not active in sport at T1, the Intervention and Control groups were equally likely to report sport activity at T3, $X_{\left(1\right)}^{2}$ = 0.10, *p* = 0.74, Cramer's V = 0.02; % active in sport: Intervention = 13.1%; Control = 11.1%.

##### Outcomes Measured Only at 1-Month Follow-up

With respect to *sport program registration*, the model was non-significant, $X_{\left(2\right)}^{2}$ = 2.69, *p* = 0.26, Nagelkerke *R*^2^ = 0.10. The model for *event registration* was significant, $X_{\left(2\right)}^{2}$ = 10.71, *p* < 0.001, Nagelkerke *R*^2^ = 0.11, but “Group” did not significantly contribute to variance in registration (*p* = 0.27, *B* = 0.58).

## Discussion

### No Significant Differences Between the Two Experimental Groups

Directly after exposure, participants in the GFM-alone and GFM+SE groups reported similar OEs and were equally as likely to seek a sport-related newsletter. After exposure and at the 1-month follow-up, both groups reported similar intentions to do sport, barrier SE, scheduling SE, and similar MVPA levels. Participants in both experimental groups also reported similar rates of sport participation, and registration in sport programs and events. Thus, contrary to our hypotheses, there was no advantage for recipients of the additional efficacy-enhancing messages.

Although the efficacy-enhancing messages in the paired condition embedded mastery and vicarious SE sources, tailored directly to barriers for adults, they did not increase barrier/scheduling SE compared to the GFM-alone condition. Since 64.3% of GFM+SE participants were *not* active in sport at baseline, it is possible the messages intended to enhance efficacy may have actually reinforced the belief that there are barriers to adult sport participation. Wegner (1994) Theory of Ironic Processing posits that while a conscious thought process directs attention toward a desired behavior (e.g., sport participation), parallel “ironic” processing monitors indicators of potential failure (e.g., lack of ability to overcome barriers). Ironic processing is more automatic, less effortful, and can cause unwanted thoughts/behaviors when an individual is facing high cognitive demand. The cognitive demand of completing survey measures may have led to ironic processing; “efficacy-enhancing” messages may have paradoxically caused some participants to focus on potential failure of *not* overcoming barriers, negating any advantage from GFM+SE.

Barrier efficacy-enhancement may not be the most appropriate approach to pair with a messaging intervention when attempting to stimulate PA, at least not in the context of adult sport. Pairing GFM with other protocol may be more effective. Lithopoulos and Young (2016) paired GFM with an identity-elaboration (“possible sport selves”) protocol and found benefits for enhanced sport-specific information-seeking behavior and sport registration, and significant increases in sport intentions. Sweet et al. (2014) paired GFM with messages designed to increase motivation for action planning and found that higher quality action plans were developed among participants in the paired condition. Having participants deliberately write about aspects of a “possible sport self” or create an action plan for PA engages individuals to a greater extent than simply reading promotional messages.

### Comparing the Collapsed Experimental to the Control Condition

#### Psychological Outcomes

The results provided insight about the effects of receiving *either* messaging intervention (i.e., GFM+SE or GFM-alone) compared to a control condition. The Intervention group consistently trended positively in reports of OEs over time, while the Control group remained constant or slightly decreased in their reports of anticipated OEs. Specifically, the Intervention group showed significantly stronger effects for the anticipated likelihood that sport would result in travel opportunities, opportunities to make friends, and opportunities for stress relief. The largest increases over time pertained to the likelihood of adult sport to provide travel opportunities and opportunities to make new friends. Whereas traditional messages promoting PA have often focused on widely-acknowledged health and fitness benefits, travel and social affiliation opportunities via sport may be more novel for adults, and therefore made a greater impression on the participants (Petty et al., 2009). *OE-Travel* may have seen the greatest increase among Intervention participants because it had the greatest potential for growth, as it had the lowest baseline mean of the OEs.

The many non-significant between-group results indicate that the messages were perhaps not strong enough to elicit changes in key psychological variables, or that the one-time exposure to the intervention was insufficient to significantly affect most variables (Latimer et al., 2008). It is likely that reports of OEs increased because the nine GFM specifically targeted beliefs about positive outcomes of adult sport participation, which were congruently reflected in each OE item. Although a few OEs were enhanced among experimental participants, this advantage did not appear to translate into gains in other important outcome cognitive variables (e.g., intentions). Beliefs about the benefits of sport are important in the early stages of behavior change, but additional psychological antecedents like SE and intentions should also be enhanced to actually increase behavior (Cavill and Bauman, 2004). Additional messages (or alternative sport-promotion tactics) that specifically target other key cognitive antecedents should continue to be explored to understand how to best motivate sport behavior.

#### Behavioral Outcomes

The frequency by which Intervention and Control participants requested a sport-related newsletter was similar immediately after exposure, as was their weekly sport behavior and sport registration 1-month later. There was a notable trend whereby MVPA levels significantly decreased from T1 to T3 for the Control group, but scores among the Intervention participants did not drop. As we collected the majority of data from November to January (predominantly winter months in Canada), participants were possibly subjected to seasonal declines in MVPA. The non-intervention Control group perhaps reflected the seasonal downturn, whereas Intervention participants may have benefitted from receiving a sport-promotion messaging intervention that “buffered” against seasonal declines. This said, this inference about sport messaging having a buffering effect on diffuse (non-sport) types of PA is admittedly tenuous (results were shy of a significant interaction for MVPA in the total sample of participants) and will need to be proven in future research. Our conclusion is that our messaging interventions designed to specifically promote sport activities did not stimulate increases in sport-specific behavior. Past messaging studies have also reported difficulty in changing PA behavior (e.g., Latimer et al., 2010); our results reinforce the challenge of motivating increases in targeted PA behaviors through messaging.

#### Limitations and Future Directions

This study had limitations that could be improved upon in future work. There was a high rate of attrition, which has been cited in many web-based interventions (e.g., Wangberg et al., 2008). Thus, our sample could not be targeted to the extent that has been recommended by messaging researchers (e.g., tighter age group, only individuals in early stages of change; Latimer et al., 2010; Lithopoulos et al., 2015). There remains a need to implement tactics to retain more participants in these types of interventions.

Attrition analyses allowed for better understanding of differences between those who dropped out and those who adhered throughout. Firstly, individuals assigned to an experimental condition were more likely to drop-out than those in the Control group, suggesting the added participant burden of watching a promotional video may be to blame. Secondly, individuals were more likely to complete the entire study if they had lower expectations regarding the beneficial outcomes of adult sport prior to intervention, and if they were not active in sport as an adult or during youth. Thus, it is possible that individuals that were already active in sport “turned off” this sport-promotion study, as they were sufficiently motivated to be engaged in sport activities without a messaging intervention. This explanation may also explain why adults who reported higher OEs at baseline, or those who participated in sport in youth, were more likely to drop-out. Drop-outs may have already been convinced of the benefits of sport and did not see any use for remaining within the study.

Our sport-promotion messaging intervention was not sufficient to stimulate significant increases in the majority of our outcome variables. Research should continue to investigate more effective ways to enhance key psychological and behavioral outcomes related to adult sport. Messaging literature has shifted away from providing cognitive/instrumental information (e.g., logical reasons why participation in PA is beneficial that would typify traditional GFM) to more affective-based information. For example, Sirriyeh et al. (2010) showed how people who received PA-promotional text messages targeted to affective beliefs (e.g., enjoyment) increased their PA more than those who received a set of instrumental belief messages, those who received combinations of affective and instrumental belief messages, and a control group. Recent messaging interventions for PA have documented advantages for messages focusing on affective beliefs and outcomes over cognitive-based rationale (Morris et al., 2016; Ruissen et al., 2018). Since our messages contained primarily instrumental-based information, future work could investigate the effectiveness of affective-based messages in sport.

Messaging may be more effective as part of a more holistic approach. For example, considering sponsorship/recruitment by a significant other who is already involved in adult sport is a key conduit for attracting new participants (Stevenson, 2002), tying messaging to an immediate protocol that links recipients to possible mentors/sponsors (who share similar personal attributes) may hold promise. Another direction is to deliver targeted and tailored messages about sport to personal devices (Mistry et al., 2015) or to provide persuasive messages to individuals already participating in a behavioral intervention (i.e., a program; Kinnafick et al., 2016).

## Data Availability

The datasets generated for this study are available on request to the corresponding author.

## Ethics Statement

This study was carried out in accordance with the recommendations and full ethics approval from the Health Sciences and Sciences Research Ethics Board at the University of Ottawa, Office of Research Ethics and Integrity. Written informed consent was obtained from all subjects. All subjects gave written informed consent in accordance with the Declaration of Helsinki.

## Author Contributions

ML initiated the ideation, design, and execution of this study. She led analyses, interpretation and discussion of results. BY supported ML in a supervisory role and provided feedback at all stages of research, including write-up and edits.

### Conflict of Interest Statement

The authors declare that the research was conducted in the absence of any commercial or financial relationships that could be construed as a potential conflict of interest.

## Footnotes

^1^Both Tversky and Kahneman (1981) and Rothman and Salovey (1997) discussed how prospective gain-framed messages may be received differently depending on whether prospective gain-frame outcomes are viewed as certain or as risky, i.e., not certain or encumbering some risk in the pursuit of gain-frame conditions. This interaction of gain-frame messaging and risk has received little attention and we did not set out to address this interaction explicitly in the current experiment. However, it was important to screen for risk to ensure (a) our targeted audience for the intervention did not see the criterion behavior as inheriting undue personal health risk, and (b) that there were non-significant differences between our experimental groups at baseline.

^2^Although the SQUASH self-report procedure is purported to derive continuous data, inspection of our returned data showed highly non-normal distributions across the entire sample, and within groups. This was likely because the SQUASH asks people to list activities in a drop-down box and then, for each activity, to report commensurate scores in boxes to estimate frequency and intensity of engagement in that activity. In essence, an individual's insertion of a categorical variable is multiplied by scaled indices for frequency and intensity to arrive at a SQUASH score. When no categories are inserted representing sport, then there is no multiplication, and the distributions take on a bi-modal pattern, with one end of the distribution showing a prevalence of zero scores. At the other end of the distribution, for those who inserted one or more sport activities, there is greater variability of sport activity data. This led to the decision to score participants data as ‘yes’ or ‘no’ to sport activity.

^3^To confirm our results were replicated with the smaller cohorts that reported being “healthy enough” to regularly participate in sport, all analyses were re-run with only those participants in each group. Results were the same for the healthy-enough sample, as were effect sizes, with two exceptions that are identified in subsequent endnotes.

^4^The same analyses for “healthy enough” participants revealed one novel significant result for *OE-Thrills, F*_(1, 173)_ = 3.95, *p* = 0.04, $\eta_{p}^{2}$ = 0.02. Trends indicated the Intervention group had increased expectations and the Control group decreased in expectations for thrills from T1 to T2.

^5^The same analysis for “healthy-enough” participants indicated a significant group by time interaction effect for *MVPA, F*_(1, 145)_ = 4.79, *p* = 0.03, $\eta_{p}^{2}$ = 0.03. Post-hoc tests revealed that GLTEQ scores decreased from T1 to T3 for the Control group (*p* = 0.05, $\eta_{p}^{2}$ = 0.02), but scores among Intervention participants did not change (*p* = 0.32).
