# Supplementary material for: Gain-Framed Messaging for Promoting Adult Sport: Examining the Effects of Efficacy-Enhancing Information
Source: Front Psychol. 2019 Mar 8;10:431. doi: 10.3389/fpsyg.2019.00431 (PMC6419619; doi:10.3389/fpsyg.2019.00431)
Supplement: Supplementary file 1 [file Presentation_1.pdf]

Appendix A

Gain-framed messages that were presented in a randomized order

**Benefit of adult sport**	**Message**
Health and fitness	Many Masters athletes claim that their participation makes them feel **fitter and healthier**. If you get involved in Masters sport, you too can increase your aerobic capacity, strength and flexibility, and overall health.
Delay the effects of aging	Many Masters sport participants tell us that they continue to do it because it helps **delay the effects of aging**. Masters sport can give you a chance to retain a youthful look and feel.
Social affiliation	Masters sport participants tell us that it provides great opportunities for **fellowship with other likeminded individuals.** If you get into Masters sport, you too will also make many friends whom you can interact with on a regular basis.
Enjoyment	Participants in Masters sport tell us that it is a way for them to **enjoy themselves and have fun**. If you get involved in Masters sport, you too will learn to love the game again, like you did years ago.
Stress relief	Masters participants indicate that, oftentimes following training, they feel calmer and more relaxed. If you get involved in Masters sport, you too can significantly **reduce stress and tension**.
Improve physical skills	Masters sport gives people chances to **develop new physical capabilities** and to prevent existing ones from declining. If you get involved, you too will have opportunities to work on techniques and strategies in sport.
Thrills and excitement	Participants tell us that Masters sport helps prevent them from ever being bored. You too will find yourself constantly **stimulated by thrills and challenges** in training and competition.
Travel	Many participants tell us that it lets them break away from the same daily routine by offering chances for away games, tournaments, or training camps. You too will be more likely to **travel and see new places** through adult sport.
Achieve competitive goals	Masters participants tell us that they regularly have opportunities to **pursue their competitive goals**. If you take up Masters sport, you too can compare yourself against your own standards and can test your skills against others.

Note. In the preface to the video, participants were informed that adult sportspersons are often referred to as Masters athletes/participants.

Appendix B

Efficacy-enhancing messages that were presented in a randomized order

**Barrier to sport participation**	**Message**
Lack of time (interferes with other obligations)	When first thinking about joining adult sport, others **just like you** were not so sure they could find time for it because of other commitments. However, with a little bit of **scheduling and planning,** they soon found that they could do it. **You too** can find the time to get involved in Masters sport!
Lack of motivation	When first thinking about joining adult sport, others **just like you** worried that they didn't have enough drive to get involved. However, by **committing to a sport that was right for them and setting goals**, they soon discovered they could motivate themselves. **You too** can become motivated and begin participating!
Negative attitude (feeling "too old")	When first thinking about joining adult sport, others **just like you** worried that it was not for people their age. However, by **keeping an open mind and adopting a positive attitude, they tried it out** and discovered it's for people of all ages. **You too** can give Masters sport a try regardless of your age!
Lack of facilities and/or opportunities in neighborhood	When first considering participating**,** other people **just like you** did not know of opportunities for adult sport in their community. By **seeking out information**, they found the right program and facility to get involved in Masters sport. **You too** can easily find an opportunity that's close by and right for you!
Lack of encouragement from family and/or friends	When first considering taking up Masters sport, many others **just like you** felt little encouragement from family and friends. However, by **sharing with others why participating is important to them**, they were able to recruit support. **You too** can gain encouragement from others to begin participating in sport again!
